# Pregabalin and atypical NSTEMI presentation in a diabetic woman (masked myocardial ischemia): A case report

**DOI:** 10.3892/mi.2026.300

**Published:** 2026-02-04

**Authors:** David Donkor, Tatiana Rodriguez, Ruth Abebe, Parth Adrejiya, Lewam Berhe

**Affiliations:** 1 Department of Internal Medicine (Residency), WellStar Spalding Medical Center, Griffin, GA 30224, USA; 2 Philadelphia College of Osteopathic Medicine, Moultrie, GA 31768, USA; 3 Department of Internal Medicine (Faculty), WellStar Spalding Medical Center, Griffin, GA 30224, USA

**Keywords:** acute coronary syndrome, electrocardiogram, multivessel disease, myocardial infarction, pregabalin

## Abstract

Women with non-ST-elevation myocardial infarction frequently present with atypical symptoms, particularly those with diabetes, in whom autonomic neuropathy can blunt the perception of ischemic pain. The present study reports the case of a 57-year-old female patient with type 2 diabetes, chronic obstructive pulmonary disease, hypertension, hyperlipidemia, obesity and chronic pregabalin use who presented with nausea and bilious vomiting without chest pain. An initial evaluation revealed mildly elevated high sensitivity troponin T, non-specific electrocardiogram (ECG) changes and normal abdominal imaging. Acute coronary syndrome (ACS) was initially considered unlikely, and the patient was discharged following an improvement in her symptoms. However, within 24 h, she re-presented with recurrent gastrointestinal symptoms and new periumbilical pain. Repeat ECG demonstrated diffuse ST-segment depressions and T wave inversions with upright T waves in aVR and V1. A subsequent echocardiography revealed a reduced ejection fraction (25-30%), and coronary angiography confirmed severe multivessel coronary artery disease. The present case report underscores the diagnostic challenge of atypical ACS in diabetic women, highlights the critical value of serial ECGs and biomarker trending in high-risk patients, and raises awareness of gabapentinoid therapy, such as pregabalin, as a potential contributor to the delayed recognition of myocardial ischemia. Early, repeated ECG evaluations should be prioritized even in the absence of chest pain to prevent the missed or delayed diagnosis of life-threatening coronary disease.

## Introduction

Acute coronary syndrome (ACS) may present with a wide spectrum of symptoms, and the absence of chest pain is well recognized, particularly among women and patients with diabetes. Diabetic autonomic neuropathy can blunt the perception of ischemic pain, leading to atypical manifestations, such as nausea, vomiting, abdominal discomfort or generalized weakness, rather than classic substernal chest pain. These atypical presentations are associated with a delayed diagnosis, suboptimal treatment and worse clinical outcomes, contributing to the higher mortality rates observed among women with myocardial infarction (1-4).

Electrocardiography remains a cornerstone in the evaluation of suspected ACS, and dynamic changes on serial electrocardiograms (ECGs) are critical for detecting evolving ischemia, particularly in non-ST-elevation presentations (5,6). Certain ECG patterns, including diffuse ST-segment depressions and T wave inversions with upright T waves in aVR and V1, have been shown to be associated with severe multivessel or left-main equivalent coronary artery disease and carry significant prognostic implications (7).

Gabapentinoids, including pregabalin and gabapentin, are commonly prescribed for diabetic neuropathy; however, their use has been linked to adverse cardiovascular outcomes and may further obscure the recognition of ischemic symptoms in high-risk patients (8-10). The present case report describes a case of atypical ACS in a diabetic woman receiving pregabalin, ultimately found to have severe multivessel coronary artery disease.

## Case report

A 57-year-old woman with type 2 diabetes mellitus, hypertension, hyperlipidemia, obesity, chronic obstructive pulmonary disease, arthritis, migraines, and a 40-pack-year smoking history presented to the Emergency Department of Wellstar Spalding Medical Center (Griffin, GA, USA) on September 27, 2025 with sudden-onset nausea and bilious vomiting without chest pain. She reported constipation and generalized weakness. Her home medications included pregabalin at 300 mg three times daily.

**Figure 1:**
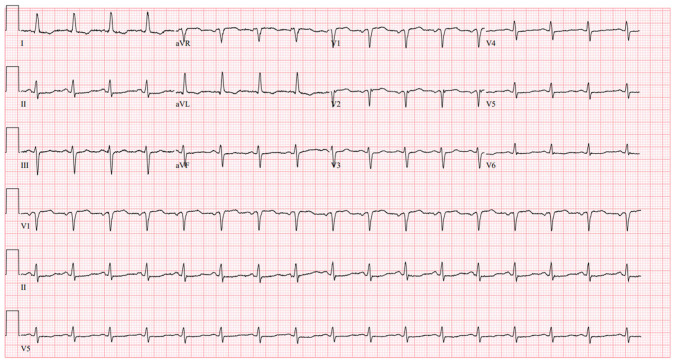
Electrocardiogram performed at the initial presentation of the patient. This revealed sinus rhythm, low-voltage QRS and non-specific ST-T changes.

**Figure 2:**
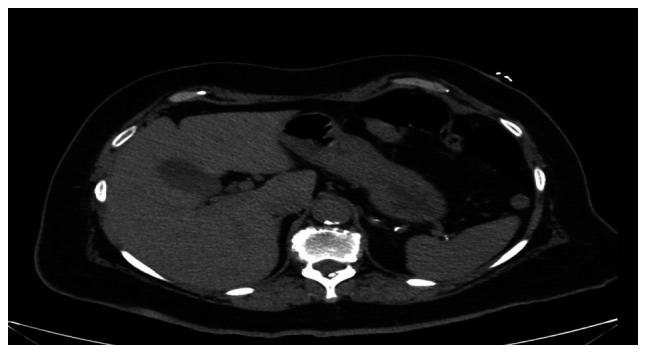
Non-contrast computed tomography scan of the abdomen demonstrating no acute intra-abdominal abnormality.

An initial evaluation revealed mildly elevated, yet rising high sensitivity troponin T (103 → 129 → 161 ng/l), non-specific ECG changes characterized by low-voltage QRS complexes and T-wave flattening in V4-V6 (Fig. 1) and a normal chest radiograph; an abdominal computed tomography (CT) scan (Fig. 2) did not reveal any notable findings. Despite the elevated levels of cardiac biomarkers, the absence of chest pain and improving gastrointestinal symptoms led to a working diagnosis of non-cardiac etiology. She received aspirin (81 mg), statin therapy (atorvastatin, 80 mg), β-blocker (metoprolol succinate, 100 mg) and a heparin drip for 48 h and supportive care and was discharged following presumed stabilization.

**Figure 3:**
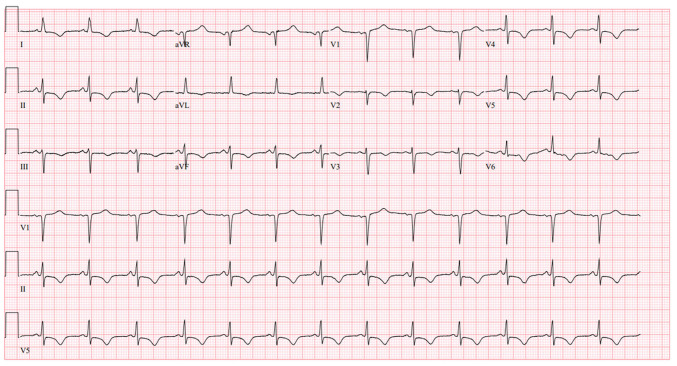
Electrocardiogram performed upon the readmission of the patient. This revealed ST-segment depressions and T-wave inversions in the inferior (II, III and aVF), lateral (I, aVL and V5-V6), and anterior (V2-V4) leads, with upright T waves in aVR and V1.

However, within 24 h, the patient returned with persistent nausea, vomiting and new dull periumbilical pain. A repeat ECG demonstrated diffuse ST-segment depressions with T-wave inversions in inferior (II, III and aVF), lateral (I, aVL and V5-V6) and anterior (V2-V4) leads, along with upright T waves in aVR and V1 (Fig. 3). High sensitivity troponin T was down-trending (~64 ng/l). Her thrombolysis in myocardial infarction (TIMI) score was 5, and the Global Registry of Acute Coronary Events (GRACE) score was 103. Transthoracic echocardiography revealed a severely reduced left ventricular ejection fraction (25-30%) and grade I diastolic dysfunction.

**Figure 4:**
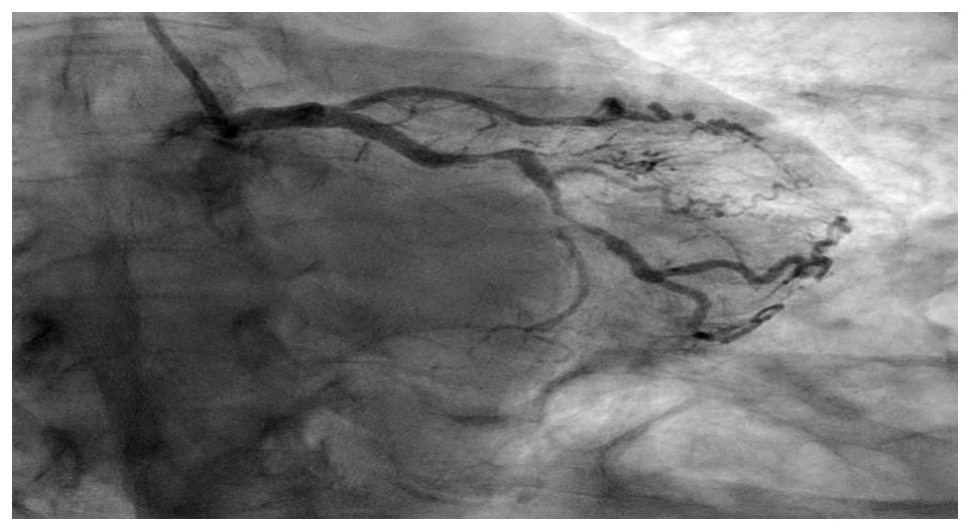
Coronary angiography of the right coronary artery (RCA) demonstrating reported proximal 60-70% stenosis, with additional reported 20-30% stenosis in the mid segment and mild distal disease.

**Figure 5:**
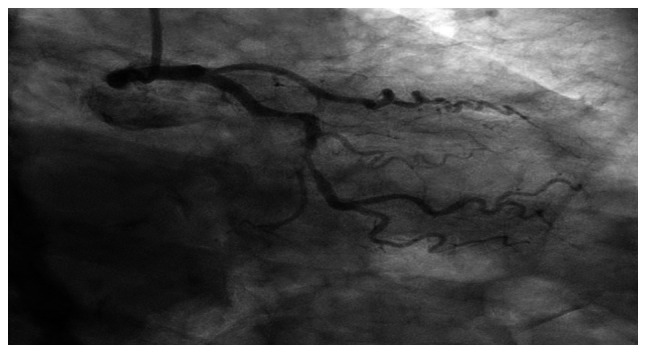
Left circumflex coronary angiography showing mild proximal disease with reported severe mid-circumflex stenoses (80-90% and 70-80%), with obtuse marginal branches demonstrating mild disease, as reported.

**Figure 6:**
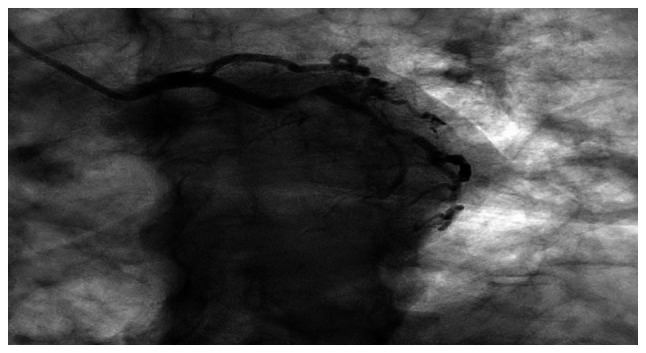
Coronary angiography of the right coronary artery demonstrating reported 20-30% stenosis in the posterior descending artery (PDA) and a reported focal 90% stenosis in the posterolateral (PL) branch.

Coronary angiography confirmed severe multivessel coronary artery disease, including the chronic total occlusion of the left anterior descending artery with collateral flow from the right coronary artery, severe mid-circumflex stenosis (80-90%) and proximal right coronary artery stenosis (60-70%) with distal posterolateral branch stenosis (90%) (Fig. 4, Fig. 5 and Fig. 6). Left ventricular end-diastolic pressure measured at 12 mm Hg, and anterior wall hypokinesis was noted. She was managed with guideline-directed medical therapy, including aspirin, 81 mg; clopidogrel, 75 mg; metoprolol succinate, 100 mg; atorvastatin, 80 mg; lisinopril, 40 mg; sublingual nitroglycerin, 0.4 mg; and a heparin drip for 24 h and referred for cardiology evaluation and potential revascularization. She underwent evaluation for coronary artery bypass grafting and subsequently underwent coronary artery bypass graft surgery. She was discharged on post-operative day 5. Her pre-operative left ventricular ejection fraction improved from ~30% to 41-45% post-operatively. At the 4-week follow-up, she was clinically stable and cleared to begin cardiac rehabilitation and return to work as tolerated.

## Discussion

Women and patients with diabetes frequently exhibit atypical symptoms during ACS, including nausea, vomiting, abdominal discomfort, or generalized weakness, rather than the classic substernal chest pain. Diabetic autonomic neuropathy may blunt the perception of visceral pain, altering sympathetic and sensory responses and contributing to atypical presentations (1-4). These clinical variations often lead to delayed or missed diagnoses, resulting in worse outcomes and higher mortality among women compared with men (2,4).

In the case presented herein, the initial presentation of the patient with gastrointestinal symptoms led to a diagnostic focus on abdominal pathology, despite elevated troponins and cardiovascular risk factors. The absence of chest pain, coupled with apparent improvement following supportive therapy, created a false sense of reassurance that contributed to her premature discharge. This emphasizes a critical clinical lesson: In high-risk individuals, particularly in diabetic women, a single ECG or biomarker assessment is insufficient to exclude ACS. Instead, serial ECGs and troponin trending are indispensable, even when initial findings are non-diagnostic or symptoms transiently improve, in alignment with consensus recommendations for non-ST elevation ACS evaluation (5,6).

A critical turning point occurred during the return visit of the patient, when a repeat ECG revealed diffuse ST-segment depressions and T wave inversions, with upright T waves in aVR and V1 (Fig. 3), a pattern strongly associated with severe multivessel or left main equivalent ischemia (7). Such global subendocardial changes often indicate widespread coronary insufficiency and should prompt urgent cardiologic evaluation. Notably, her troponin level was declining at this point, underscoring that reliance solely on biomarker trends may overlook evolving ischemia; electrocardiographic monitoring, rather than biochemical data alone, proved decisive in establishing the true severity of her condition (5).

An additional consideration in the case described herein involves the chronic use of pregabalin by the patient. Diabetic autonomic neuropathy is a well-established and likely primary mechanism underlying painless or atypical myocardial infarction in patients with diabetes, due to blunted visceral pain perception and altered autonomic signaling (1-4). Gabapentinoids, such as pregabalin and gabapentin, are widely prescribed for diabetic neuropathy and chronic pain, and have been increasingly linked to adverse cardiovascular events, including myocardial infarction and heart failure (8-10). Proposed mechanisms include fluid retention, autonomic modulation and effects on vascular tone, which may further elevate the threshold for ischemic symptom perception. In this context, pregabalin may have functioned as a contributory or exacerbating factor, amplifying the blunting of symptoms already present due to diabetic neuropathy, rather than serving as the sole cause of the masked ischemic presentation. Clinicians should therefore remain vigilant when evaluating atypical symptoms in high-risk patients receiving these agents (8-10).

In conclusion, the present case report reinforces several key diagnostic principles. First, the atypical presentations of ACS, particularly in women with diabetes, should not diminish clinical suspicion (1-4). Second, symptomatic improvement should never preclude serial cardiac evaluation when risk factors are present. Finally, repeat ECGs, rather than a single initial tracing, may reveal dynamic, high-risk patterns indicative of multivessel coronary disease. In the patient in the present study, the diagnostic turning point was not imaging or laboratory normalization, but rather the recognition of evolving ischemic changes on serial ECG evaluation (5-7). Maintaining a low threshold for ECG repetition and troponin trending in high-risk populations, even in the absence of chest pain, is therefore essential to avoid premature diagnostic closure and prevent missed or delayed identification of life-threatening coronary disease (5,6).

## Data Availability

The data generated and/or analyzed during the present study are available from the corresponding author on request.
